# Perceived behavioral control, intention to get vaccinated, and usage of online information about the human papillomavirus vaccine

**DOI:** 10.1080/21642850.2013.869175

**Published:** 2014-01-21

**Authors:** Rebecca Katherine Britt, Kristen Nicole Hatten, Scott Owen Chappuis

**Affiliations:** ^a^ School of Communication, The University of Akron, 110K Kolbe Hall, E. Buchtel Avenue, Akron, OH44325, USA; ^b^ Brian Lamb School of Communication, Purdue University, 100 North University Street, Lafayette, IN47907, USA

**Keywords:** HPV vaccine, theory of planned behavior, perceived behavioral control, moderation analysis

## Abstract

*Objective:* Human papillomavirus (HPV) and the HPV vaccine have been examined through multiple lenses over the past several years, though there is little work examining the role of perceived behavioral control (PBC) and its impact on potential recipients retrieving, understanding, and using online information with regard to the vaccine. *Method:* This study used survey data to examine the role of PBC as a moderator between attitudes and intention, and subjective norms (SN) and intention to get the HPV vaccine; and PBC as a moderator when seeking out Facebook, YouTube, and Twitter as information sources about HPV. Support was found for each prediction. *Results:* The interaction term of SN × PBC in particular had a strong influence on intention to get the vaccine. *Discussion:* Planned behavior variables explain intention to get the HPV vaccine, but data also suggest a need to root this research within intervention-based strategies.

A wide body of research in the past several years has examined the human papillomavirus (HPV) vaccine. The sorts of advertising and educational messages which appeal to the target demographic – high school- and college-aged women – have been analyzed, indicating that there can be a sexually transmitted disease-related stigma attached to obtaining the HPV vaccine (Friedman & Shepeard, 2006). There is also a concern among some parents that vaccinating their adolescent might lead to the adolescent engaging in sexual behavior (Brewer & Fazekas, 2007). Additionally, those in rural areas are much less likely to be aware of the vaccine or its effectiveness toward preventing cervical cancer and other related complications (Cates, Brewer, Fazekas, Mitchell, & Smith, 2009). In other words, there is an uphill battle facing programs and campaigns that are attempting to encourage young women to use the HPV vaccine.

HPV vaccine programs have been effective when emphasizing high vaccine effectiveness, likelihood of HPV infection, and recommendations from physicians (Brewer & Fazekas, 2007). Additionally, when viewed through a gain- and loss-frame perspective, individuals who engaged in low-frequency risk behaviors were more likely to react positively (e.g. to feel motivated to obtain the vaccine) when faced with loss-frame messages (Gerend, Shepherd, & Monday, 2008). Despite the interest in HPV and the HPV vaccine, there is still little work done examining the role of perceived behavioral control (PBC) and its effect on potential recipients retrieving, understanding, and using online information about the vaccine.

It is well known that PBC was added into the original iteration of what is now the theory of planned behavior (TPB) (Fishbein & Ajzen, 1975). TPB added PBC into the model to account for when people intend to behave in a particular way, but the actual behavior changes because of a lack of self-efficacy (SE) or control over that behavior. TPB is a popular framework and potentially more robust than other theories when explaining understanding, knowledge, and intention to get the vaccine. Furthermore, scholars have addressed a need to examine TPB in a more nuanced fashion when examining sensitive health issues, such as HPV (Yzer, 2007).

This article addresses the construct of PBC and its relationship to attitudes and (SN) with intention to getting the HPV vaccine. Additionally, we look at the role of information-seeking behaviors relating to HPV, particularly through social media. Specifically, we look at the role of PBC as a moderator between attitudes and intention, and subjective norms (SN) and intention when seeking out Facebook, YouTube, and Twitter as information sources about HPV.

## Literature review

The TPB has been used previously to study patients' and parents' intentions to vaccinate and attitudes toward the vaccination. There is, however, a gap in the literature when addressing if and how patients utilize online resources in their search for more information on the vaccination. TPB was developed by psychologist Ajzen (1991). According to TPB, human action is guided by three kinds of considerations: beliefs about the likely outcomes of the behavior and the evaluations of these outcomes, known as behavioral beliefs; beliefs about the normative expectations of others and motivation to comply with these expectations, known as normative beliefs; and beliefs about the presence of factors that may facilitate or impede performance of the behavior and the perceived power of these factors, known as control beliefs (Ajzen, 2002). Central to TPB is the individual's intention to perform a given behavior. Intentions are assumed to capture the motivational factors that influence a behavior; they are indications of how hard people are willing to try, of how much of an effort they are planning to exert, in order to perform the behavior (Ajzen, 1991).

### Planned behavior research on the HPV vaccine

In recent years, a large effort has been made to promote uptake of HPV vaccine, which can cause cervical cancer and genital warts (Bryan, 2007). Two vaccines (Gardasil and Cervarix) are approved for use by the American Federal Drug Administration (FDA) (Centers for Disease Control and Prevention, 2010). Each of the vaccines requires a three-dose regimen with one to five months between doses, depending on the particular vaccine; adherence to this regimen was typically high (Einstein et al., 2009). However, there are barriers that stand in the way of people receiving the vaccine in the first place. One barrier can be the acceptance of parents and adolescents or young adults with regard to the vaccine, but it can also be the burden of the healthcare provider. It has been argued that providers are hesitant to recommend the vaccine to younger adolescents and strongly desire the endorsement of a professional organization prior to recommending the vaccine to their patients and patients' parents (Zimet, 2005). Another barrier is perception of risk; if individuals do not feel they are at risk for contracting HPV and related diseases, they are less likely to get vaccinated (Brewer & Fazekas, 2007; Wong, 2008). Additionally, the cost of the vaccine proves prohibitive for some patients (Keating et al., 2008; Wong, 2008).

In particular, much research has been devoted to examining factors associated with female intention to receive the vaccine (Boehner, Howe, Bernstein, & Rosenthal, 2003; Gerend & Magloire, 2008; Jones & Cook, 2008; Krawczyk et al., 2012). Intention to receive the HPV vaccine is linked to doctor's recommendations (Jones & Cook, 2008), perception of greater benefits and few barriers such as cost (Juraskova, Bari, O'Brien, & McCaffery, 2011), and perceived susceptibility (Gerend, Lee, & Shepherd, 2007). Planned behavior research has suggested that positive attitudes toward the HPV vaccine and influence of significant others, such as peers and parents, are consistently associated with intention (Allen et al., 2009; Kahn et al., 2008; Ogilvie et al., 2007).

College women remain a targeted population for good reason; they are largely susceptible to HPV due to demographic factors (Centers for Disease Control and Prevention, 2010). Current research finds that TPB variables account for as much as 60% of the variance accounted for in intention to get the HPV vaccine, even when testing TPB against other models, including the Health Belief Model (Bennett, Buchanan, & Adams, 2012; Gerend & Shepherd, 2012), social cognitive theory (Bennett, Buchanan, & Adams, 2012), and the predecessor to TPB, theory of reasoned action (Roberto, Krieger, Katz, Goei, & Jain, 2011). Mothers of college-age daughters were examined through the lens of TPB and found to have very low intention of vaccinating their daughters; what intention was there was significantly influenced by attitudes and subjective norms (SN) (Askelson et al., 2010).

What the current body of research does *not* address is the relationship of the construct of PBC to attitudes and subjective norms (SN) with intention to getting the HPV vaccine. Furthermore, we propose that the construct of PBC is a moderator between attitudes and intention, and subjective norms (SN) and intention, when seeking out Facebook, YouTube, and Twitter as information sources about HPV and the vaccine. Social media is a unique means by which individuals can learn more about the HPV vaccine. Facebook, Twitter, and YouTube are some of the most important social media forms that individuals use when they want to learn about these vaccines. The term itself, “social media”, has yet to have a concurrent definition used in research, but Merriam-Webster's (2013) recent definition is one that we take to be helpful in examining the intention to use social media to learn about HPV. That definition states that social media, “[are] forms of electronic communication through which users create to share information, ideas, personal messages, and other content, as videos” (para 1). Technologies such as social media are a means by which individuals can learn more about their health and develop questions for their physicians. These social media sites act as soundboards, allowing individuals to share their thoughts, concerns, and opinions about the vaccines. While many of the individuals who comment may not be experts about the vaccine, their feedback provides a useful insight for the individuals who make the decision of whether or not to get the vaccine.

## Planned behavior variables of interest

The planned behavior variables of interest include: attitudinal beliefs, normative beliefs, and PBC, the latter of which we will explore as a moderator of attitudinal and normative effects on behavioral intention.

### Attitudinal beliefs

Attitudes are the sum of beliefs about a given behavior and weighed on the evaluation on those beliefs. Attitudes and norms are not weighed equally in predicting behavior, due to individual nuances in attitudes and beliefs. A person who cares little for what others think about their intention to get the HPV vaccine would have little use for subjective norms (SN).

### Normative beliefs

Subjective norm (SN) is a central social influence factor a person's perception that most people important to them think they should or should not perform a behavior in question (Fishbein & Ajzen, 1975). Typically, norms are conceptualized as injunctive norms referring to rules/beliefs that determine what constitute approval or disapproval in conduct. Typically, an injunctive norm is about social sanctions – what should be done – if an action is not carried out. Subjective norms (SN) are mainly concerned with a person's perception of others' important expectations for a given behavior, but injunctive norms are more connected to social disapproval.

Subjective norms (SN) influence behavior through motivation to comply with referents' normative beliefs and expectations. Additionally, norms have a direct effect on behavioral intent through compliance or coercive social pressure.

### Perceived behavioral control

PBC is essentially the same idea as SE; the measure of a person's perception of his or her ability to complete a task. What differentiates PBC from SE, however, is that PBC incorporates two specific aspects: perceived capacity (PC) and perceived autonomy (PA) (Yzer, 2007). (PA) is the degree to which one believes they can perform a behavior; (PA) is the degree to which they believe they have control over the actual behavioral performance (Yzer, 2007). (PC) and (PA) tend to converge; Armitage (2005) found that degree of confidence and degree of control in engaging in physical activity were highly correlated. Some research has only found moderate support for PC and PA, such as with breast self-exams (Norman & Hoyle, 2004) and giving blood (Giles, McClenahan, Cairns, & Mallet, 2004).

Ultimately, PBC is concerned with the resources and opportunities that either encourage or hinder behavioral performance. As such, the present study looks at PBC in the context of confidence and certainty of being able to complete a given task (Yzer, 2012).

### PBC as a moderator

According to traditional research on reasoned action, attitudes, perceived norms, and PBC affect behavioral intent in a direct method, but even if attitudes and perceived norms toward a behavior are positive, if people lack the SE to do so, then they will not engage in that behavior (Eagly & Chaiken, 1993; Yzer, 2007). Thus, PBC may moderate attitudinal and normative effects on behavioral intention. When the relationship of attitudes and norms is strongly related to intent, intention may be higher when PBC is higher (Ajzen, 2002; Yzer, 2007). This follows the idea that when a behavior is perceived to be under one's control, then attitudinal and normative effects on intention can perform better in explaining behavioral intention.

Adding PBC into the mix as a third variable may affect the relationship between attitudes and intention, and subjective norms (SN) and intention to get the HPV vaccine. As such:

H1: For sexually active adults, PBC moderates the relationship between attitudes and intention to get the HPV vaccine.

H2: For sexually active adults, PBC moderates the relationship between subjective norms (SN) and intention to get the HPV vaccine.

Given the relationship between ability to maintain sexual health and information-seeking behavior about HPV, we next look at actual information-seeking behaviors using social media – namely, Facebook, YouTube, and Twitter, which are identified as key resources that people use today to learn and communicate about information relating to HPV (Shoenfeld, 2012). Indeed, the evolution of new computerized technology and social media has had an impact on health communication (Lewis, 2010). That definition itself, combined with a body of current research, even identifies social media's intrinsic link with the intention to actively communicate to share information (Lewis, 2010; Wright & Hinson, 2010).

In Dillard and Spears’ (2010) research on perceived barriers to being vaccinated against HPV, a predictor was found in self-report frequency of media exposure. Similar research continues to advocate for the role of PBC as a moderator regarding intention to engage in positive, healthy behaviors (Amireault, Godin, Vohl, & Perusse, 2008; Kimiecik, 1992). Practically speaking, intention tends to be a significant determinant of behavior (Sheeran, Trafimow, & Armitage, 2003), and the PBC-behavior relationship has not been given attention regarding social media attitudes, which could play an important role in designing interventions toward vaccination intent. Since social media use requires the person to be active, we questioned the following:

H3: For sexually active adults, PBC moderates the relationship between attitudes and intention to use social media web sites (Facebook, YouTube, and Twitter) to learn about HPV.

## Method

### Design and participants

Upon Institutional Review Board approval, data were collected in 2012 in a suburban community in the Midwestern USA. An online survey was distributed via an e-mailed link to a university population, including both faculty, staff, undergraduate, and graduate students. The survey was created to assess students' use of the Internet to address health concerns or issues. The survey asked participants questions about their sexual health, their use of the Internet to address health issues, and their perceptions and intention to get or complete the HPV vaccine. One hundred and seventy-four participants took the survey, ranging in ages 18–54 (*M* = 22.5, SD = 5.14), and the majority of participants were undergraduate students (*M* = 2.76, SD = 1.15). Participants predominantly reported Caucasian/White as their ethnicity (86.5%), with some Asian/Pacific Islander (4.1%), African American (5.4%), Hispanic/Latino (1.4%), Undisclosed (2.6%), and 5 missing values.

In the survey, participants first answered a series of demographic questions, followed by a question that asked whether or not they have a health condition that requires regular interaction with a physician. Participants were not asked to elaborate on this answer; they only identified if they had some condition. The majority of participants did not report a condition (91.9%), but some did (8.1%; missing values = 5). After this, they answered four questions relating to how they maintain their sexual health, including whether or not they have had unprotected sex (76.8% said yes), and whether or not they feel they are good at maintaining their sexual health (1–5 point Likert Scale from “Strongly Agree” to “Strongly Disagree” and all participants reported either “Agree” or “Strongly Agree”). Next, participants responded to questions that asked them about whether or not they had received the HPV vaccine – no participants in the entire sample had received the vaccine. Finally, participants responded to their perceptions of information where they could learn about the HPV vaccine. Upon completion of those questions, participants had completed the survey and were thanked for their time.

## Measures

### Intent

Intent to get/complete the HPV vaccination was measured by the average of three items (“I intend to get/complete the HPV vaccine in the next year,” “I plan to get/complete the HPV vaccine in the next year,” and “I want to get the HPV vaccine in the next year.”) on a seven-point scale (*M* = 4.24, SD = 1.98). The scale showed high internal consistency (Cronbach's alpha = .96).

### Attitudes

Following typical recommendations for TPB research (Ajzen, 1991) attitudes toward getting the HPV vaccination were measured by the mean of an eight-item semantic differential (e.g. beneficial/harmful, good/bad, worthless/valuable, etc.) on a seven-point scale (*M* = 4.80, SD = 1.47) in response to the base question, “Getting/completing the HPV vaccination over the next year would be … ” These items showed strong reliability (Cronbach's alpha = .93).

### Subjective norms (SN)

Perceptions of normative influences over getting the HPV vaccination were measured through the average of four times (e.g. “Most people who are important to me think that I should get the HPV vaccination” and “Many women like me plan on getting/completing the HPV vaccination.”) on seven-point scales (*M* = 2.80, SD = 2.04). The scale showed good internal consistency (Cronbach's alpha = .91).

### Perceived behavioral control

The average of five items was used to assess PBC using seven-point scales (*M* = 3.73, SD = 1.24). Items included, “If I wanted to, I could get/complete the HPV vaccination in the next year,” and “It is mostly up to me if get/complete the HPV vaccination in the next year.” The items showed good reliability (Cronbach's alpha = .90).

### Attitudes toward social media use

Attitudes toward using social media sites (Facebook, YouTube, and Twitter) were measured by the mean of an eight-item semantic differential (e.g. beneficial/harmful, good/bad, and worthless/valuable) on a seven-point scale (*M* = 4.32, SD = 2.45). The items showed decent reliability (Cronbach's alpha = .79). Attitudes toward social media have been assessed in similar ways with similar reliability (Lewis, 2010; Wright & Hinson, 2010).

The dependent variable was intention to get the HPV vaccine with the item “I intend to get/complete the HPV vaccine within the next 12 months.” Participants who had already completed the HPV vaccine were excluded from the analysis. Responses were coded on a 1 to 7 Likert scale ranging from *strongly disagree* to *strongly agree*. The mean was 3.53 (SD = 1.83, *N* = 174).

### Analyses

Multiple regression analysis was used to test each hypothesis. Variables were mean-centered and product terms were created to represent all two-way interactions. The means, standard deviations, and zero-order correlations of all variables are displayed in Table 1.

**Table 1.** Means, standard deviations, and intercorrelations of studied variables.

Variables	*M*	SD	1	2	3	4	5	6	7
1. Gender	–	–							
2. Age	22.50	.526	.229	–					
3. Attitudes toward HPV vaccine	4.86	1.53	.214	−.229	–				
4. Subjective norms (SN) toward HPV vaccine	4.13	1.83	.222	−.162	.447**	–			
5. Intent to get/complete HPV vaccine	3.36	1.93	.061	.408**	.447**	.664**	–		
6. Perceived behavioral control toward HPV vaccine	3.71	1.37	.242*	.232*	.181*	.158	–	–	
7. Social media use relating to HPV vaccine	1.08	.262	−.001	.419**	.020	.001	−.074	–	–

Note: *N* = 174.

*p < .05.

**p < .01.

## Results

Dillard and Spear (2010) argued that a revised integrated model of planned behavior finds that PBC is a moderator on the effect of attitudes and subjective norms (SN) with intention to get the HPV vaccine. As such, it is necessary to explore the relationship between attitudes and PBC, and subjective norms (SN) and PBC in a more nuanced fashion. Given that there is past research that finds support for the relationship of attitude and PBC on intention and subjective norms (SN) and PBC on intention, we have evidence that suggests there is indeed an indirect effect (Hayes, 2009). Because PBC is already a moderator, as several studies have shown (Ajzen, Albarricin, & Hornik, 2007; Dillard and Spear, 2010), we conducted a moderated regression analysis to investigate the strength of the relationship on the moderated variable.

To investigate a moderating effect of PBC, we computed regressions on attitudes toward intention to get the HPV vaccine, intention to get/complete the HPV vaccine, norms relating to intention to get/complete the HPV vaccine, and PBC and intention to get/complete the HPV vaccine as predictors.

H1 stated “For sexually active adults, PBC moderates the relationship between attitudes and intention to get the HPV vaccine.” Hierarchical multiple regression was performed. The control variables (gender and age) were initially entered into step 1 and were not significantly associated with intention to get/complete the HPV vaccine, *F* (2, 139) = .250, *p* < .493, *R* ^2^ = .043. Step 2 included attitudes toward getting the HPV vaccine. In step 3, PBC toward getting the HPV vaccine was included. Finally, in step 4, the interaction term of ATT × PBC was added. Prior to regression analysis, all independent variables were centered to reduce multicollinearity errors. Table 2 shows the results of the analysis. Attitudes were significantly related to intention to get the HPV vaccine in each step – in particular, we look at the fourth step of the model, *B* = .286, SE*_B_* = .074, *β* = .222, *p* < .05, ^*R* ^2^ = .009, which includes attitudes, PBC, and the interaction term. However, we consider the baseline of *p* < .05 and the sample size. When the Attitudes × PBC term was added to the model, there was a significant relationship to the model, although it was small, *B* = .142, SE *_B_* = .166, *β* = .031, *p* < .05, ^*R* ^2^ = .001. However, there is support for H1.

**Table 2.** Hierarchical linear regression for attitudes × perceived behavioral control.

Variable	Step 2				Step 3				Step 4			
	*B*	SE*_B_*	*β*	*p*	*B*	SE*_B_*	*β*	*p*	*B*	SE*_B_*	*β*	*p*
Intercept												
1. Gender	.002	.463	−.113	.997	.073	.472	.019	.877	.080	.477	.021	.867
2. Age	−.427	.450	.386	.347	−.441	.452	−.117	.333	−.435	.456	−.115	.344
3. Attitudes toward HPV vaccine	.495	.155	.386	.002	.476	.157	.371	.004	.286	.074	.222	.034
4. Perceived behavioral control toward HPV vaccine					.139	.172	.097	.002	.044	.753	−.031	.095
5. ATT × PBC									.142	.166	.031	.033
*R*^2^ (Adj. *R*^2^)			.178	(.139)			.187	(.134)			.188	(.120)
^∧^*R*^2^				.136				.009				.001
*F*				10.25				.657				.063
^∧^*F*				.002				.421				.803

Note: *N* = 174.

H2 stated “For sexually active adults, PBC moderates the relationship between subjective norms (SN) and intention to get the HPV vaccine.” Hierarchical multiple regression was again performed; step 2 included subjective norms (SN) toward getting the HPV vaccine. In step 3, PBC toward getting the HPV vaccine was included. Finally, in step 4, the interaction term of SN × PBC was added. Table 3 shows the results of the analysis. When subjective norm (SN) was added to the model, a strong relationship was found, *B* = .628, SE*_B_* = .341, *β* = .032, *p* < .001, ^*R* ^2^ = .001. Furthermore, a stronger relationship for SN × PBC was found than ATT × PBC, which may suggest that peer influence has a stronger relationship to PBC and subsequent intention, *B* = .146, SE*_B_* = .322, *β* = .144, *p* < .014, ^∧^ *R* ^2^ = .001.

**Table 3.** Hierarchical linear regression for subjective norms (SN)  × perceived behavioral control.

Variable	Step 2				Step 3				Step 4			
	*B*	SE*_B_*	*β*	*p*	*B*	SE*_B_*	*β*	*p*	*B*	SE*_B_*	*β*	*p*
Intercept												
1. Gender	−.349	.489	−.089	.478	.314	.392	.081	.426	.308	.296	.079	.439
2. Age	−.325	.366	−.086	.516	−.333	.367	−.088	−.907	−.321	.373	−.085	.070
3. Subjective norms (SN)toward HPV vaccine	.711	.105	.665	.000	.701	.106	.657	.000	.628	.341	.032	.001
4. Perceived behavioral control toward HPV vaccine					.112	.142	.078	.000	.146	.322	.144	.014
5. SN × PBC									.018	.079	.091	.022
*R*^2^ (Adj. *R*^2^)			.454	(.427)			.460	(.423)			.460	(.414)
^∧^*R*^2^				.411				.006				.001
*F*				1.37				4.59				.623
^∧^*F*				.001				.433				.820

Note: *N* = 174.

Finally, H3 stated “For sexually active adults, PBC moderates the relationship between attitudes and intention to use social media web sites (Facebook, YouTube, Twitter) to learn about HPV.” Hierarchical multiple regression was again performed; step 2 included attitudes toward using social media web sites (specifically, Facebook, YouTube, and Twitter) to learn about the HPV vaccine. In step 3, PBC toward getting the HPV vaccine was included. Finally, in step 4, the interaction term of PBC × Social Media was added. Table 4 shows the results of the analysis. Attitudes toward relevant HPV social media contributed to the model, but greatly reduced in step 4, although the interaction term had relatively good support, *B* = .307, SE*_B_* = .043, *β* = .040, *p* < .009, ^*R* ^2^ = .031.

**Table 4.** Hierarchical linear regression for social media use regarding HPV vaccine × perceived behavioral control.

Variable	Step 2				Step 3				Step 4			
	*B*	SE*_B_*	*β*	*p*	*B*	SE*_B_*	*β*	*p*	*B*	SE*_B_*	*β*	*p*
Intercept												
1. Gender	−.263	.508	−.067	.606	−.127	.514	−.032	.806	−.187	.523	−.047	.045
2. Age	−.927	.520	−.239	.080	−1.00	.519	−.259	−.050	−1.15	.564	−.299	.045
3. Attitudes toward social media about HPV	.375	.058	.086	.008	.528	.587	.657	.018	.031	.915	.007	.007
4. Intention to use social media about HPV					.270	.196	.182	.008	−.059	.503	−.039	.117
5. PBC × social media use									.307	.043	.040	.009
*R*^2^ (Adj. *R*^2^)			.054	(.005)			.084	(.020)			.093	(.020)
^∧^*R*^2^				.007				.031				.031
*F*				.417				1.90				.505
^∧^*F*				.521				.173				.480

Note: *N* = 174.

## Discussion

In this study, we elaborated on research done on the HPV vaccine using a planned behavior framework (Ajzen, 1991). We found support that suggests that perceived ability to actually get and complete all three shots required of the vaccine moderates the relationship between attitudes toward the vaccine and actual intention to get the vaccine. More specifically, we argued that when reordered, treating PBC as a moderator resulted in an interesting theoretical finding that suggests that Dillard and Spear's (2010) proposed integrated model is indeed a viable option for future planned behavior research, particularly with respect to the ordering and structuring of variables like PBC. This has particular implications for HPV research, which in recent years has sought to explicate factors underlying behavioral intention (Centers for Disease Control and Prevention, 2010; Friedman & Shepeard, 2006; Go, 2010).

H1 and H2 suggested that when added to the model as an interaction term, PBC would moderate the relationship between attitudes and intention to get the HPV vaccine and subjective norms (SN) and intention to get the HPV vaccine. Support was found for both hypotheses, although we found a greater relationship for the impact of subjective norms (SN). Despite this contribution, its limitations should be noted. The results of this study are survey based, which, while useful, are somewhat limiting for H3, which looked at the role of social media content related to the HPV vaccine. We included this hypothesis because numerous studies have recommended exploring online content about vaccine information-seeking behaviors (Roark, 2011), and given the accolade for PBC as a moderator, this was an exploratory opportunity. While there was support for H3, we acknowledge that in order to fully make this claim, researchers should more closely examine this content. Experimental designs integrating HPV social media could lend a greater insight into what content appeals to users and how that content can better address user needs.

## Contribution of PBC in changing health behavior

This study was rooted in examining one of the most widely used psychological theories (TPB) in HPV vaccination behavior. Moreover, Dillard and Spear (2010) paved the way for using PBC (i.e. barriers in deciding not to obtain the HPV vaccine) as a moderator. We replicated this theoretical contribution from Dillard and Spear to see if perceived barriers made a meaningful contribution when attitudes toward using social media were considered. Our results were similar; we found that perceived barriers to getting the HPV vaccine influenced whether or not individuals would actually engage in that behavior. We found that perceived barriers moderated the relationship with the intention to use social media to learn about HPV, which is particularly interesting because, as Dillard and Spear (2010) argue, “encouraging more realistic assessment of the risk of HPV … could translate into greater vaccine acceptance” (p. 190). Yet, just as important, this also means that while attitudes toward social media regarding HPV could be important, if there is a lack of perceived control in being able to get the vaccine – and perhaps knowing just what social media platforms to use – then nothing really changes. We argue that this is a first step toward a concerted need to design an intervention that does three things:

1. An intervention design for HPV vaccination intention can be done in a way that is firmly rooted in a planned behavior approach focusing on the strength of control beliefs (Ajzen & Sheikh, 2013) to predict behavior using pre- and post-assessment.
2. Performing a behavior can have both positive and negative consequences, and concerns regarding the HPV vaccine are rampant, which therefore heighten the need to develop strong facilitating factors. Social media can be especially useful serving in this capacity by stimulating positive beliefs through the use of graphics, video content, narrative statements, and more. Increasing control beliefs in this content will surely help.
3. Finally, an effort in an intervention needs to be made to encourage conversation about the HPV vaccine. Perceived control may be related to avoidance (Ajzen & Sheikh, 2013) in some health behaviors, so it would make sense that using social media not only to share materials, but also to facilitate conversation, will be an added benefit in stimulating positive change (Roark, 2011).

Indeed, numerous possibilities exist for examining perceived control, but it is clear that a strong intervention should be a next step in the research. As a key predictor of behavior, intention is important to study, but the gap between intention and behavior may be better understood through PBC, which can contribute to the prediction of behavior and help to inform intervention design.

## Limitations and future directions

The current study used an online survey based on convenience sampling. This was a useful first step in gathering data necessary to begin to explore the behavior–intent relationship surrounding the HPV vaccine, but it is a notable limitation. For instance, studying populations both younger and older would provide very valuable data and an additional insight. Furthermore, a limitation of the current study is that with social media still being measured in many ways, we chose to measure it in a way similar to Ajzen's (1991) recommendations of attitudes. Other studies, like Lewis (2010) and Wright and Hinson (2010), chose to measure attitudes toward social media with items using phrasing such as “How important are each of the following [blogs, electronic forums, etc.] … ” which, while helpful, could capture something other than attitude. As a study firmly rooted in TPB, we measured attitudes toward social media in a way similar to measuring attitudes toward the HPV vaccine. Future studies can overcome this in a number of ways, most notably by (1) seeking to create a measure of attitudes toward social media that includes intention toward usage and statements that point toward previous and perception of future usage and (2) firmly creating an intervention that uses social media as the primary mechanism for content delivery, and also capture just what social media is. As we see in the current dictionary definition (Merriam-Webster, 2013), there is a lack of consensus on the definition, which is certainly a limitation for researchers and contributing to a better understanding of the subject matter.

Research on planned behavior has long suggested that perceived control can moderate the relationship between attitudes and intention, but the support of these data suggests that there may be a deeper need to continue to examine PBC in further HPV research, and for research beyond health issues. If PBC is a persistent factor in deciding to get the HPV vaccine, then perhaps it should be more aptly targeted in intervention-based research. Furthermore, HPV has been a widely addressed issue in recent years, with a range of studies focusing anywhere from young adult to older adults, female and male intention to get the vaccine, and so forth. HPV in particular is an interesting issue to address using an integrated planned behavior model because the outcome of behavioral intention is very direct. The results from this study can also have an impact on public health policy and intervention; knowing how individuals use social media and its potential effect on their attitudes and intentions toward getting a vaccine can help public health policy-makers in designing campaigns to reach particular populations.

Our results also showed that PBC moderated the relationship between attitudes and intention to use social media web sites to learn about HPV. In this context, perceived control refers to one's perceived ability to seek out and make use of materials about HPV found through social media web sites. Perceived control again emerged as a significant factor, which speaks to the power of social media, and that we can further utilize it as a platform to target in HPV research.
